# Exploring our relationship with nature: A modified-Delphi study protocol

**DOI:** 10.12688/openreseurope.16295.1

**Published:** 2024-05-30

**Authors:** Cassandra Murphy, Tadhg MacIntyre, Elaine Gallagher

**Affiliations:** 1 Department of Psychology, Maynooth University, Maynooth, County Kildare, Ireland; 2 ALL Institute, Maynooth University, Maynooth, County Kildare, Ireland; 3 TechPA Research Group, Inland Norway University of Applied Sciences, Hamar, Norway; 4 Insight SFI Research Centre for Data Analytics, Maynooth University, Maynooth, County Kildare, Ireland; 5 SPAICE Consulting, Bristol, UK

**Keywords:** Nature Connectedness, Nature Relationship, Biophilia, Expertise, Interviews, Qualitative, Delphi, Scale Development

## Abstract

**Background:**

A vital element to understanding the the health and wellbeing of both humans and the environment is human-nature interactions. The biophilia hypothesis is referred to when discussing these interactions. This hypothesis suggests that due to evolution, humans have an innate urge to seek out nature. The concept of nature connectedness was developed from this hypothesis and is rooted in the belief that human identity and nature can be intertwined. This research aims to explore the intricate details of how an individual builds this connection in a meaningful way.

**Methods:**

This is done using a modified Delphi method. A Delphi study in its typical form aims to gather the consensus of a group of experts in a specific area of interest. This modified Delphi aims to break down the barrier between the public and the experts by creating a second category of participants referred to as our ‘expanded experts.’ Expand experts are described as individuals with lived experience of being connected to nature in the everyday. This category comprises of artists, city planners, activists and many more. This allows for a much more inclusive and real-world exploration of experiences. The participants will first take part in a semi-structured interview process to investigate their experiences of connecting with nature. Following a hybrid thematic analysis with both deductive and indictive coding will be applied to the interviews. These themes will be shared with participants for them to weigh the importance of the theme to the construct to allow a deeper understanding of our interactions with nature.

**Results:**

The results of this project will contribute to and shape the development of a state-of-the-art nature-connectedness scale. Furthermore, understanding how nature connectedness fits into our modern world will allow for more appropriate nature-based interventions for urban residents and beyond.

## Plain language summary

This interview-based study looks at the interaction’s individuals have with the nature around them and how those interactions help them to feel connected to the natural world. Interviews are taking place with fellow researchers, as well as individuals who work with nature in their everyday. Some examples are nature-based therapists and practitioners, poets, artists, farmers and city planners to name a few. By undertaking this research, it is hoped that we will better understand what nature resources urban residents require in their areas to improve their overall health and wellbeing as well as how individuals interact with the resources the already have access to. The end goal is to produce a method to measure these interactions for future research and practical use.

## Introduction

The term nature connectedness (NC), which can also be referred to as connectedness to nature and nature relatedness, is typically used to describe the human-nature relationship, and the positive emotional, physical and cognitive connection between humans and their environment ( Barrable & Booth, 2022). Baxter and Pelletier (2019) have expanded on this construct to consider nature connectedness to be a psychological need, supporting Wilson’s (1984) biophilia hypothesis which proposed that our urge to engage with nature was innate. This finding strengthens the argument that high levels of nature connectedness are linked to the overall wellbeing of humanity ( Keenan *et al.*, 2021; Nisbet *et al.*, 2011) and the environment ( Gosling & Williams, 2010; Jensen & Olsen, 2019), which highlights the importance of understanding this connection. Recent conceptualisations have postulated that there are five pathways to connect with nature: contact, emotion, compassion, meaning and beauty ( Lumber *et al.*, 2017). Support for this pathways model has emerged from the ‘three good things’ intervention which reported in general that noticing things in nature can increase nature connectedness scores ( Keenan *et al.*, 2021). However, this kind of intervention may have limited impact in an urban setting, where high-quality natural areas are typically unavailable. Differences in scores on nature connectedness have also been reported when it comes to both age and gender. In children it has been found that girls are more connected than males, despite boys reporting having more contact with nature ( Mustapa *et al.*, 2021). Among adult student samples no gender differences were found, however some personality factors, which may have developed over time and into adulthood, may be responsible for this change ( Di Fabio & Rosen, 2019). This raises the question as to how broad our current understanding of nature connectedness is, as it only focuses on positive experiences, and ignores negative experiences labelling those who score low on the existing measures as disconnected, rather than exploring other factors such as preference, experience or mode of nature exposure ( Barrable & Booth, 2022; Salazar *et al.*, 2021).

### Gaps in literature

A recent review ( MacIntyre *et al.*, 2019, p.21) has described nature connectedness as “a blurred construct with a range of competing concepts developed in order to describe the human-nature relationship, including connectedness to nature ( Mayer & Frantz, 2004), and environmental identity ( Clayton, 2003).” This variety in understanding the overall construct has led to disparity among measures with Salazar and colleagues (2021) highlighting the inconsistency and lack of efficacy and usability of these measures for practitioners on the ground. Similarly, there is a need for conceptual clarity in how nature connectedness is conceptualised.

### Aims and objectives

The aim of this study is to examine the meaning of nature connectedness in the 21 ^st^ century context (e.g. with increased urbanisation, digitalisation, coronavirus) and to address some of the aforementioned limitations of the current definitions around the construct of nature connectedness. This study will explore the following questions:-

1. Determine whether there a distinctive developmental dimension to the construct?
2. Is nature connectedness trait-like or does it have state-like components?
3. What are the steps individuals take to reach a sense of nature connectedness?
4. Does urban nature require a distinct component within the overall construct?
5. Does digital nature require a distinct component within the overall construct?
6. To what extent can advances in the construct be clearly articulated within an overall framework.

The findings will be used to advise on the development of a novel scale to assess the relationships formed between humans and the natural world, as part of another study in the GoGreenRoutes H2020 project.

## Protocol

### Study design

A modified-Delphi approach will be applied as this method has been previously employed to successfully elicit knowledge on complex constructs and ultimately achieve consensus among experts ( Fink-Hafner *et al.*, 2019; Taylor, 2020; Trevelyan & Robinson, 2015). The Delphi method is often used when there in limited or conflicting information on a topic ( Taylor, 2020). Data collection for a Delphi technique is in the form of questionnaires which are answered by a panel deemed as experts, usually I the form of academics or medical professionals ( Hsu & Sandford, 2007). The modification of the Delphi method in this study is two-fold: 1) A semi-structured interview process will be utilised to elicit comprehensive knowledge about the construct and the lived-experiences of participants, and 2) the sample includes two panels, the experts and the expanded experts, will be used to gain consensus. The same panel will be used for both the interview and weighting process of the Delphi method. The steps taken in the modified-Delphi method are outlined in Table 1. The interviews in this research are phenomenological in nature as it prioritises the lived experience of the participant in the questioning ( Groenewald, 2004).

**Table 1.** Systematic steps of the modified-Delphi study design.

Step 1	Elaborate selection criteria created for experts and expanded experts
Step 2	Make expert panel and recruitment strategy for expanded experts
Step 3	Contact both experts and expanded experts and confirm participation
Step 4	Conduct Semi-Structured Interviews
Step 5	Transcribe interviews and return transcripts for approval and redaction by participant
Step 6	Conduct deductive analysis.
Step 7	Conduct inductive analysis.
Step 8	Administer weighting questionnaire Round 1.
Step 9	Analyse Data.
Step 10	Administer weighting questionnaire Round 2.
Step 11	Analyse Data
Step 12	Synthesise results and publication.

## Methods

As outlined in Table 1 and above, the study will use a semi-structured interview method to augment the traditional Delphi rounds. Recent research has shown that saturation in qualitative research can occur between 7 and 9 interviews ( Hennink & Kaiser, 2022). This research aims to have a minimum of 9 participants from each panel (expert and expanded experts) and a maximum of 30 participants overall, as a means to avoid saturation. Research has shown that the average number of respondents in a Delphi study range from 15–20 ( Hsu & Sandford, 2007) which allows room for participants who will drop-out between rounds.

A semi-structured interview schedule ( MacIntyre *et al.*, 2019) will be designed based off of previous literature on the topic of nature connectedness, and will be refined based upon the results of two pilot interviews, one from each target panel. This will allow for flexibility in each interview and allow for deeper discussions around specific concepts the participant may be an expert in.

After providing informed consent online via Qualtrics (an online survey platform), preceded by an information sheet with the opportunity for participants to submit questions to the researcher about the process of the study, the participants will be asked to complete a short pre-interview survey. This short survey is used to gather demographic information to determine their suitability for the study, alongside information on their current level of nature connectedness (NR-6; Nisbet & Zelenski, 2013) and their pro-environmental behaviour (Self-Report Index of Habit Strength; Verplanken & Orbell, 2003). They will then be asked to take part in a semi-structured interview with a researcher which will be recorded (via video if online or through audio if in person. Interviews will be conducted in English. The details of the study will be explained verbally to the participants before they begin the interview, allowing them to ask questions or withdraw their consent if they should wish to. The interviews will be initially transcribed using Otter.ai but will be manually reviewed and checked for errors before analysis. In the weeks following the interview, the participants will be asked to review the transcript of their interview and to approve the quotes, which will be coded to remove any identifying information.

A hybrid thematic analysis approach will be taken in relation to the analysis of this data ( Fereday & Muir-Cochrane, 2006; Swain, 2018). As part of the initial analysis a round of deductive coding will be conducted to allow for the item generation for the scale development study (separate study) to begin. The researchers will meet and discuss the possible codes for this analysis. These codes will be based off the questions asked in the interviews. These questions were developed based off the current literature on the topics of nature connectedness and urban nature. This will then be followed by a deeper indictive coding to uncover some of the new and complex ideas in the data which will uncover the construct.

Following the thematic analysis of the interviews, participants will be asked to take part up to three rounds of questionnaires referred to as Delphi rounds. In these separate rounds the participants will be asked to ‘weight the importance’ of various items. The items will resemble the themes which will be developed as a result of the thematic analysis. These different Delphi rounds will take the form of three separate 10–15-minute online surveys in which participants rate (often referred to as weighting the importance of statements related to nature connectedness on a five-point Likert scale from ‘very limited importance’ to ‘very important’. There will be a 4–6-week time period between each round to give participants time to complete the task. Reminder emails will be sent every two weeks in the 6-week period to promote full participation. The different rounds are used to:

1. Build consensus
2. Resolve differences
3. Prioritise information.

The information gathered from the weighting rounds will be used to develop the construct of nature relationships.

### Selection criteria for experts and expanded experts

 The participants will comprise two samples:

1. Experts: Researchers, predominantly in environmental psychology and environmental science and allied areas, who have studied the concept of nature connectedness. This will include researchers in psychometrics who developed inventories, those who have employed measures in empirical research and those who have conceptualised on this topic and related areas (e.g., human-nature interactions).
2. Expanded Expert Group: This additional group will comprise individuals for whom a connection with nature is central to their work in literature and the creative arts, in architecture and urban planning, or in addressing one of a variety of aspects relating to natural systems.

### Recruitment

The expert panel will be recruited through a systematic search for papers using the keywords ‘nature connectedness’, ‘nature relatedness’, and ‘human nature relationship’. Using software such as Scopus and researchrabbit.ai, a list of authors who have published in these areas will be collated and contacted. For the expanded-expert panel a social media recruitment campaign will be used to find interested parties. This will require potential interested participants to complete a Microsoft Forms document with basic identifying information giving the researchers permission to follow up with them. Snowball sampling will also be used, whereby a participant can forward on information to relevant colleagues who may be interested in participating in the research.

**Anonymity** – In certain cases it is difficult to keep participants completely anonymous in studies, especially when working with individuals who are considered experts in their field, such as the participants in this study. As this is a conversational style interview there may be segments of individuals’ discussion which can link them to their work, or whose thoughts and ideas may be considered intellectual property. To protect the participant the study offers an option to waive anonymity, allowing them to have their sentiments associated with their identity. Participants will also be offered the opportunity to review their transcripts and given the option to remove sections which they believe to be too identifying or change their decision to waive their anonymity based on having seen the results of the interview.

**Right to withdraw consent** – It was decided that due to the nature of a Delphi study participants cannot withdraw consent once the first round of the weighting activity has begun. This is because the information they provide in the interview is used to develop the weighting rounds. Participants can withdraw consent “until a point” – this point being when they approve their transcription. They are free to withdraw from participation, however their data will still be used. This will be acknowledged when participants sign a consent form and will be repeating for clarity upon receiving their transcription to review at which point, they are free to withdraw.

## Materials

Interviews will be recorded using Voice Memo software (in-person interviews) or MS Teams Classic Version 1.5.00.22362 (online interviews). Audio files will be saved in a m4a format with Otter.ai used for automated transcription. Following the automated transcription, the transcripts will be manually checked and edited for accuracy. MAXQDA, a qualitative coding software will be employed, or the alternative Taguette may be used. Subsequent weighting activities will employ Qualtrics Survey Platform (RRID:SCR_016728) or Google Forms (RRID:SCR_023174) with IMB SPSS Statistics Software (RRID:SCR_016479) used for analysis or the open access alternative R Project for Statistical Computing (RRID:SCR_001905). Access to licenses for the proprietary software is granted through Maynooth University.

### Data collection

The research will be conducted either online or in person depending upon participant location and availability.

### Data analysis

The analysis takes place in three steps which includes the thematic analysis, completed using both deductive and inductive coding ( Fereday & Muir-Cochrane, 2006; Swain, 2018), and the quantitative analysis.

1. The deductive coding will be based off the work of Crabtree & Miller (1999).
2. The indictive coding following the recent guidelines set out for reflexive thematic analysis, see Braun & Clarke (2022) for steps.
3. For the quantitative elements of the research the main statistical analyses that will be conducted are descriptive statistics such as central tendency (percentage of agreement, mean, median and mode), and level of dispersion (e.g., interquartile range, range, standard deviation, coefficient of variance).

## Discussion

The research findings will be invaluable to overcome the definitional dilemma with the array of terms used to operationalise nature connectedness. This potential impact will be important for applied researchers and practitioners in understanding human nature interactions more clearly. This research will allow us to gain more insight into the stages of building a connection with the natural world, including elements of technology and the urban environment.

## Ethics and dissemination

Local research ethics committee approval was granted for this study (SSREC approval no. 2441635). The findings of this study will be further disseminated through peer reviewed publication and digestible content for the GoGreenRoutes website and accompanying social media. The results of this study will also inform the development of a new measure based on the forthcoming construct. The results of both studies will be presented at the end of project event in Belgium, June 2024.

## Data Availability

No data are associated with this article.
