# Supplementary material for: Exploring our relationship with nature: A modified-Delphi study protocol
Source: Open Res Eur. 2024 May 30;4:107. [Version 1] doi: 10.12688/openreseurope.16295.1 (PMC11190642; doi:10.12688/openreseurope.16295.1)
Supplement: Peer review history

# Reviewer report: Reviewer response for version 1

**DOI:** 10.21956/openreseurope.17592.r41147

**Published:** 2024-06-20

**Authors:** Stephanie Wilkie

**Affiliations:** 1 School of Psychology, University of Sunderland, Sunderland, England, UK

This is a well-written and reasoned protocol for the proposed Delphi study.  

 My only concern/suggestion is the authors are conducting a Delphi study with an expanded expert group that only includes people likely to have a strong connection to nature. To represent the construct as a whole, I would suggest the authors consider whether there are additional groups to recruit from for whom nature is not central to their work. Psychometric methods typically require items that clearly differentiate people; and I have some concerns that any measure produced with be limited in that regard.  

 The rationale also plays into a narrative that everyone must be connected to nature (if not, it's a bad thing). I agree with the authors the construct is blurred, as are many others in person-environment research; so I do commend the aim of the proposed study (and its ultimate aim to create a measure). In future, it will be important to consider the convergent and divergent validity of that measure with other related constructs like place identity, place attachment, environmental identity etc. The authors do allude to this but I thought I would mention it more explicitly as well.

Is the study design appropriate for the research question?

Partly

Are sufficient details of the methods provided to allow replication by others?

Yes

Is the rationale for, and objectives of, the study clearly described?

Yes

Are the datasets clearly presented in a useable and accessible format?

Yes

Reviewer Expertise:

Environmental psychology; place identity; built and natural environments and wellbeing

I confirm that I have read this submission and believe that I have an appropriate level of expertise to confirm that it is of an acceptable scientific standard.

# Reviewer report: Reviewer response for version 1

**DOI:** 10.21956/openreseurope.17592.r41439

**Published:** 2024-06-14

**Authors:** Alexia Barrable

**Affiliations:** 1 Division of Psychology, Sociology and Education, Queen Margaret University, Musselburgh, Scotland, UK

This is a study protocol aiming to come to a much-needed consensus on nature connectedness, a term that describes a positive human-nature relationship. 

 The protocol is clear and well presented, covering all the methodological and ethical aspects of the study to be undertaken well. 

 One thing I would love to see a little more on is the proposed qualitative analysis justification: more specifically, why the authors are suggesting  deductive analysis is followed by inductive, and not the other way round. 

 Overall, however, this is a scientifically sound and ruinously designed protocol that should produce a solid study with useful results.

Is the study design appropriate for the research question?

Yes

Are sufficient details of the methods provided to allow replication by others?

Yes

Is the rationale for, and objectives of, the study clearly described?

Yes

Are the datasets clearly presented in a useable and accessible format?

Yes

Reviewer Expertise:

nature connectedness, early childhood

I confirm that I have read this submission and believe that I have an appropriate level of expertise to confirm that it is of an acceptable scientific standard.
